# Ethical erosion in newly qualified doctors: perceptions of empathy decline

**DOI:** 10.5116/ijme.57b8.48e4

**Published:** 2016-09-06

**Authors:** Emily C. Stratta, David M. Riding, Paul Baker

**Affiliations:** 1 John Radcliffe Hospital, Oxford, UK; 2 The University of Manchester, UK; 3 Health Education England (North West Office), UK

**Keywords:** Medical education, ethics, patient-centred medicine, qualitative research, empathy

## Abstract

**Objectives:**

This study sought to understand whether UK Foundation doctors perceived the phenomena of ethical erosion and empathy decline during their initial period of clinical practice, and if so, why this occurred.

**Methods:**

This qualitative study used semi-structured interviews with nine doctors in their first year of clinical practice at Royal Bolton Hospital, UK. Participants were invited to discuss the definition of empathy, how individuals acquire and maintain empathic ability, perceptions of ethical erosion in the self and others, and how clinical experiences have influenced their empathic ability. The interviews were transcribed, and analysed to identify emergent themes.

**Results:**

Each participant reported a conscious acknowledgement of empathy decline in their own and their colleagues’ early clinical experiences as doctors. Stressful working environments, the prioritisation of patients’ physical rather than psychological well-being, and the attitudes of senior colleagues were all suggested as possible causes. Some doctors believed that specialties with reduced patient contact had a culture which precluded empathy, and influenced their own practice. In addition, some described how their value judgements of patients had affected their ability to empathise. However, all doctors perceived that empathy skills were desirable in senior clinicians, and some believed that educational interventions may be useful in arresting ethical erosion.

**Conclusions:**

Newly qualified doctors are aware of ethical erosion in themselves and their colleagues as they begin clinical practice. This has serious implications for patient care. Improving working conditions may reverse this trend. Empathy skills training within undergraduate and postgraduate curricula may be a useful intervention.

## Introduction

In 2013 the Francis Report suggested that the attitude of tolerance towards poor care at Mid-Staffordshire Hospital in the United Kingdom (UK) would not have persisted ‘if those adopting it were constantly to place an empathy for the predicament of patients at the forefront of their mind’.^1^ This lack of empathy was identified as a contributing factor to a culture of incompetence that threatened patient safety and confidence in the medical and allied professions.  In contrast, there is evidence that doctors who empathise have higher patient satisfaction, reduced malpractice claims, and higher levels of clinical competence.^2^^-^^5^

The phenomenon of ethical erosion, where empathy and sympathy declines with increasing clinical experience, has been described in US medical students transitioning from pre-clinical to clinical training, and onwards into specialism and independent practice.^5^^-^^9^ Furthermore, attitude scores towards patients’ social issues decline as US students progress through medical school.^10^

Several studies have sought to understand this trend. Workplace stress has been cited as a cause of ethical erosion amongst doctors, and increasing levels of clinical responsibility and fatigue secondary to workload are possible drivers of empathy decline.^5^^,^^8^^,^^11^^-^^14^ Clinicians working in more technical specialities tend to empathise less,^8^^,^^15^^-^^17^ which can be learned from role models in more senior positions.^18^^,^^19^ It remains unclear whether this is a ‘nature’ or ‘nurture’ phenomenon or a combination of both. Psychiatrists tend to display higher empathic ability than surgeons, who spend less time interacting with patients, particularly in sub-specialties where inpatient stay for elective work is relatively short.^15^^, ^^20^

In stressful work environments, doctors and medical students may objectify their patient’s predicament as a source of humour, which may impede feelings of empathy and encourage contempt.^18^^-^^21^ Value judgements based on the patient’s socioeconomic status, the aetiology and nature of their illness, and the doctor’s perceptions of their similarity to the patient, can all affect empathy.^12^^,^^19^^,^^22^

Strategies to prevent ethical erosion have been suggested. Students with higher empathic ability at the start of their course are less vulnerable to progressive decline,^7^ and teaching programmes can be delivered to maintain ethical standards.^23^ In addition, the importance of clinical role models in prompting empathic behaviour has been recognised.^24^ However, despite calls for qualitative investigation of the phenomenon within the context of care,^25^^,^^26^ there have been no such studies undertaken in the UK.

This study seeks to understand whether Foundation doctors have perceived the phenomena of ethical erosion and empathy decline in their own practice, or in their colleagues’ practice. In addition, those doctors confirming a perceived empathy decline will be encouraged to explain why they think this decline occurs. Foundation doctors are best placed to offer a deeper understanding of why empathy declines, as they have recently moved to a position of much greater clinical responsibility where the decline may be most acute. Understanding the drivers of empathy decline empowers educators to design interventions that could mitigate the phenomenon, and any associated cost to patients. Selection processes, curricula or workplace cultures and practices could be reconfigured to ensure that standards of patient care are not compromised. Although international healthcare education, training and work practices are highly variable, there are common themes, and empathy decline in non-UK health systems has been widely reported.^27^^-^^30^ Accordingly, the results may be of value to international educators who are seeking to arrest empathy decline in their healthcare education system.

## Methods

### Study design

We conducted a qualitative study using semi-structured interviews, coding and thematic analysis. Although quantitative measures of empathy have been validated, this study sought to understand if and why Foundation doctors perceived that empathy declined during early clinical practice. Qualitative data allows a deeper understanding of those perceptions by empowering the interviewee to discuss their experiences and thoughts in greater detail.

### Participants

All 39 Foundation doctors in their first year of clinical practice at Royal Bolton Hospital received an email inviting them to participate in the study. Nine doctors gave their consent to participate and proceeded to be interviewed. The existing evidence suggests that empathy decline is most observable as greater clinical responsibility is acquired. Accordingly, we believed that newly qualified Foundation doctors with relatively little clinical experience, but enough to have observed and considered empathy decline, were the most appropriate group to participate. We argue that senior consultants are much more comfortable and stable in their work practice, and therefore less likely to be considering or experiencing empathy decline.

Ethical approval was granted by the Health Education North West and Royal Bolton Hospital research ethics committees. All participants provided written confirmation of their informed consent, and were offered copies of the resultant papers and presentations.

### Procedure and data collection

The lead investigator conducted face-to-face interviews with all nine participants. No one else was present during the interviews. The interview format was semi-structured in order to facilitate exploration of interviewee perceptions of a broad set of ideas relevant to the topic. A standardised set of questions was developed prior to the first interview to ensure coverage of the key issues arising from the literature review. This included defining empathy, how individuals acquire and maintain empathic ability, perceptions of ethical erosion in the self and others, and how clinical experiences have influenced their empathic ability. Within the semi-structured format, both parties were given the opportunity to explore the issues and ideas that arose during the discussion. No further invites were distributed following the interviews as saturation of themes had been achieved.

### Data analysis

Each interview was recorded digitally, and processed verbatim by a professional transcriber not otherwise involved in the study. Transcripts were anonymised, though the interviewer was aware of the identity of each participant.  Prior to data collection, a preliminary index of expected themes was developed by the group. The transcripts were interrogated with reference to this list, but any new or unexpected themes were also noted. Copies of the transcript for each interview were provided to each author to analyse independently. Following the content analysis, the group met to define and discuss and agree on the emergent themes.

## Results

### Participant demography

Nine doctors between the ages of 23 and 27 years gave consent, and were subsequently interviewed during their second four month rotation. Six were female, 3 were male, of which six were graduates of Manchester Medical School, with the remaining three graduates of Newcastle, Bristol and Edinburgh medical schools.  The mean duration of the interviews was 41 minutes 1 second (range 31:42 to 54:49).

### Defining empathy

Although all the participants indicated they had some conceptual understanding of empathy, most were unable to precisely define its meaning. Some spoke metaphorically of ‘putting yourself in their shoes’ (Participant numbers 2,3 and 9) whilst others cautiously attempted more literal definitions:

“Empathy is being able to familiarise yourself with a patient and try and see what it’s like to be in their shoes, try and understand what a patient feels. It is quite difficult to properly define empathy but I think it’s just about trying to understand things from the patient’s perspective more than anything.” (Number 9, male) 

The participants were also asked to define sympathy and compare it to empathy. Most found it challenging to do so:

“I would say sympathising is less of an emotional response.  I think that sympathising is providing words of comfort, providing time to someone, … they are very similar but for me there is a small difference in that you don’t have the same sort of emotional impact on yourself, you are not feeling what the other person is feeling.” (Number 1, male)

“…sympathy is seeing what someone else is feeling something whereas empathy is like being able to reflect that emotion.” (Number 4, female)

### Is empathic ability acquired or innate?

The quality of being empathic was perceived by all participants as a fundamentally innate characteristic, usually well-established by the time the individual enters the profession, and often secondary to childhood environment and early social experiences:

“I think by the time you start medical school you’ll have developed whatever empathy you are going to get and while you can sort of expand on that you’re not going to get much more, it’s something you learn from the age of four or five when your parents are bringing you up, if they’re quite caring, things like that, you’re going to pick up those traits and become quite caring yourself.” (Number 1, male)

Despite this, some commented that undergraduate training may help to enhance the student’s ability to focus on empathy, and to view it as an essential component of patient care:

“It is a skill that can be taught …well, not necessarily just taught but refined as well, I think everyone has a certain amount of empathy, some more than others, and I think it should be encouraged that whenever a healthcare professional is talking to a patient or a relative it is important to empathise with them so we can understand them better and help them better as well.” (Number 9, male)

However, others disagreed, and perceived attempts at empathy teaching to be unsatisfactory:

“They taught us ‘here is your Empathy Phrase, you stick that in and you’re being empathetic,’ but that’s not what empathy is, saying that.  It is a hard thing to teach, communication skills are a good place to start, but just saying that doesn’t mean you’re feeling it, and I think empathy is the feeling behind the words.” (Number 5, female)

### Perceived value of empathy in clinical practice

The participants demonstrated both positive and negative views of empathy. In the context of the former, empathic ability was associated with skilled and effective doctor-patient communication:

“When you’re breaking bad news to someone you have to try and think about how they are going to be feeling so you can tailor what you say to them.  And try to explain things to them try and understand their state of mind and how they’re feeling…” (Number 5, female)

Indeed, it was perceived that the act of being empathic may sometimes be the most useful intervention, particularly in patients with challenging diagnoses, such as chronic pain syndrome:

“Empathising with her, acknowledging that she had pain, helped … she left (because) she’d wanted reassurance and a bit of empathy, and a bit of understanding and some explanation as well.  (This) made her feel better, that we were listening and trying to understand what was going on, even if we couldn’t tell her why she had the pain.” (Number 3, female)

It was also argued that ‘if you can’t empathise with someone you are not going to be able to understand their behaviour’ (Participant number 3), and that failure to so deprives the patient of the holistic care required to treat illnesses exacerbated by emotional or other psychological factors. However, whilst all participants identified empathic ability as a desirable characteristic, many expressed concerns that imbalances in empathy may adversely affect clinical judgement:

“It’s possible to take too much on board and then become affected by decisions you are making. A clinically rational decision becomes too involved because there’s other factors coming into play… just because someone’s empathetic doesn’t mean they are going to make the right decision for a patient, it just means that how they communicate with them and go about making the decisions might be a little bit more patient-orientated.  Whereas less empathetic doctors might make decisions based on recent articles or knowledge and less so on the patients’ choices” (Number 6, female)

“If you empathise too much with someone and become emotionally attached then it can be difficult to make a fully objective decision.” (Number 8, female)

There was also concern that doctors who are ‘too empathic’ may begin to consider it as a threat to their mental health, with increased levels of anxiety and stress:

“I just thought ‘how hard it must be for them, they’ve come to a foreign country, they don’t speak a word of the language, and they’ve got this terminal diagnosis now.  How do I explain it to them, this is such awful news?’  So I spent the weekend worrying, because I knew that … there wasn’t anything more that I could have done but the implications, implications for them, for the diagnosis, for the family.  So that was a hard one.” (Number 9, male)

“It does (affect me) because I’m having to watch people work through very difficult emotions… and it’s difficult… I’m fine in work, when I go to the Unit, people pass away and I’m fine, I don’t cry … but then I find that once every two or three months I’ll just start crying and I’ll cry for hours, and then I’ll fall asleep for a bit, and the next day when I wake up it’s as if I’ve got rid of it, and then I start again.” (Number 6, female)

### Ethical erosion in practice

Most participants in the study identified the phenomenon of ethical erosion in their own practice. One observation was that with time and increased clinical experience the perceived emotional impact of patients and their predicaments diminished:

“Before (starting) medicine you saw someone with cancer and had a really high level of sympathy, but now you hear about some cancers think ‘it’s not a bad one!’(It’s a combination of) increased knowledge, that not everything that is called cancer deserves that amount of sympathy and we have become immune to it, we’ve just developed a high level of tolerance to illness.” (Number 5, female)

“Things probably affected me a little bit more when I started the job, I think you slowly just get used to it being a part of your everyday and so as it becomes the norm, it doesn’t distress you as much.” (Number 8, female)

In addition, ethical erosion within colleagues’ practice was also identified:

*“Generally people empathise less as they go along their career, I think they get hardened and so used to the situations* *that that becomes* *normal, if you see people dying every day it becomes normal.” *(Number 8, female)

“In surgery you sometimes feel that the seniors or the surgeons are not as empathetic, they just want to get the patient better and out, they don’t want to spend much time with the patient…” (Number 9, female)

However, in contrast, one participant valued a high level of emotional involvement in a senior colleague’s consultation:

“I’ve seen consultants cry breaking news to relatives, and speaking to patients, and I think actually that can help the patient and the family because they can see we understand how you feel and can recognise how difficult this is and we are here to help you.” (Number 6, male)

### Perceived causes of empathy decline and effect on patient care

All participants were able to suggest environmental, cultural and social factors that may affect doctors’ ability to empathise with patients. Most interviewees found it easier to identify reasons why empathic ability might decline, rather than how it could be maintained.

One commonly cited perception was that the opportunity to spend time with patients was limited. Participants perceived that the high number of patients and tasks charged to them precluded the development of more satisfying doctor-patient relationships, and impeded opportunities to empathise:

“My ability to empathise has decreased because I spend less time per patient. As a medical student you have all the time in the world to explore everything about them but you just can’t do that now.” (Number 7, female)

“…the busier you are, the easier it is to think ‘it’s just a person in a bed, it’s just a body in a bed,’ and you forget that they’re actually a person and you have to take time to just stop and think ‘I’d best go speak to them and find out about them, what’s going on with them, how they feel.” (Number 6, female)

This is closely linked with the interviewees’ perceived level of stress. Many observed that their ability to empathise declined when the workplace pressure upon them increased, and although they still valued the importance of empathy, they felt that the working environment inhibited them from being empathic:

“[Stress] is a major factor because you don’t have the time to sit down and you find yourself thinking ‘oh I just don’t have the time to do this right now’, I know it’s important but I just can’t, I need to prioritise [clinical care over empathising with patients].” (Number 3, female)

“In times when everybody’s stressed, with too much workload and there’s understaffing, it’s easy to forget empathy with your patients, it’s easy to be very one-track with treating everybody the same, and actually you need empathy to know their situation, treat them according to their situation.” (Number 7, female)

Inter-specialty variation in empathic behaviour was also observed. Senior clinicians in surgical specialties were perceived to display less empathy than their non-surgical peers:

“I am on paediatrics at the moment and the difference is phenomenal, the surgeons will spend about three seconds with a patient, they just get the key facts and then go, they don’t spend as much time with the patients getting to know them.” (Number 1, male)

“I think a lot of the time with surgeons, probably because they’ve seen so much of it, their tolerance goes up, they always see people in pain, all day every day, the majority of surgeons, abdominal surgeons, so it’s just another person with pain.” (Number 5, female)

However, there remained an appreciation that the workload of different specialties continued to impede clinicians’ opportunity to be empathic:

“On surgery, they have such pressures on to get the operations done and get their numbers up that they sacrifice other things for that; I think that’s probably an organisational thing there…they are very much like ‘I want to get into theatre rather than see patients’.  It depends what you enjoy, if you enjoy speaking to people or you really enjoy theatre.” (Number 5, female)

“…if I saw the same patient as a GP then I would be happy to spend a long time going through their concerns, talking them through it, building up a relationship with the patient, whereas in A&E I am much more likely to be ‘I can’t do anything for you, go and see your GP.” (Number 4, female)

There were examples related by the interviewees that went further, with one doctor recalling how they had been encouraged to avoid speaking to patients so that new illness would not be discovered, and would not compromise the rapid turnover of patients characteristic of surgical practice:

“I would rather not speak to them [patients] in the first place to find things out, rather than have to ignore it … ‘don’t find out he’s got a chest infection … don’t start him on antibiotics for his chest,’. I don’t know if that’s empathy … I think it’s their (surgeons) lack because they are not thinking about the patient and what’s really going on with them.” (Number 5, female)

When discussing the pressures of clinical practice, one participant reported the routine use of humour by healthcare staff as a protective tool against potentially harmful emotional engagement with patients:

“You very quickly develop a way of talking about the patients in a way that, although you are empathetic and sympathetic, protects you from being too emotionally involved, so you have a little bit of a laugh and a joke, either about the patient or the situation, or the things that are going on. As long as it’s not malicious I think it can be helpful to the working environment. It’s a way of normalising a situation, taking some of the emotions out of it.  It’s definitely a coping mechanism.” (Number 2, male)

This was developed by a different interviewee, who described the use of inappropriate humour during a ‘breaking bad news’ conversation with a critically ill patient’s spouse:

“A wife’s husband was in intensive care and wasn’t very well, then the wife was asking the consultant ‘do you think my husband will pull through?’  To which the consultant replied ‘Well I wouldn’t take his clothes to Oxfam just yet.” (Number 6, female)

Another theme that emerged during the interviews was the participants’ self-awareness and reflection of their own position within society, and how they viewed their patients’ predicaments within this context. One interviewee linked her ability to empathise with her own self-perception:

“I feel that you empathise most easily with people who are like you or remind you of, say a friend or a relative.” (Number 8, female)

Some went further, claiming that their personal value judgements of patients and their illnesses directly affected the way in which they delivered clinical care:

“I think I can be quite judgemental especially in A&E (and) in surgery as well, like people who are IV drug users or people who come in intoxicated, and people like that. So those people I probably often don’t listen to.” (Number 4, female)

“a lot of the patients were there as a result of their own lifestyle choices and so I found it more difficult to relate to them than the ones who had had these things sprung upon them, like they had done nothing wrong, and so you’d normally sort of prioritise their treatment a little bit more because you’re like ‘well this is really bad luck on your part, you didn’t know this was going to happen, you haven’t been told to stop smoking for thirty years, and then you’ve lost your legs because of smoking-related diseases.’’ (Number 1, male)

“If someone doesn’t have a serious physical illness but they’re moaning and wanting more painkillers than you think what you’ve seen deserves, it’s hard to empathise because in your opinion how they should be feeling are different from how they are communicating to you.  It’s hard to say ‘oh that must be bad’ when you are thinking ‘it’s not that bad.’’ (Number 5, female)

## Discussion

### General perceptions

This study demonstrates that some Foundation Trainee doctors in their first year of clinical practice perceive empathy decline in their own and their colleagues’ practice. Interestingly, this is not a subconscious decline. The doctors were fully aware of the attitudinal and behavioural changes in themselves and their colleagues, and appeared to view the decline as a response to the highly demanding work environment they now inhabited. Despite difficulties in defining empathy, all the interviewees were able to understand the concept in general terms, and so felt able to engage in a detailed discussion exploring ethical erosion. In most cases, an ability to empathise was seen as desirable, but was often viewed as a secondary consideration to the provision of the patient’s physical treatment.

### Possible explanations

Our data is confluent with the ethical erosion described in US medical undergraduates as they move from the classroom to the ward.^5^^-^^9^ Foundation doctors have recently completed a similar transition from the medical school to the hospital, an environmental change which is potentially stressful.^31^ In addition, they can often feel confident in their theoretical understanding of medicine, but unprepared for the daily physical and emotional demands of patient management.^32^ Foundation trainees reported feeling under pressure to ensure that all patients receive optimal physical care, at least. Some perceived that in the context of this challenging new role, they do not have the time or energy required to be empathic. Such pressures may contribute to decreased personal well-being, which has been associated with reduced empathy amongst newly-qualified doctors.^33^

For many participants, this phenomenon was particularly noticeable during their surgical placements. This is consistent with previous studies, which associate clinicians in technical specialties with lower empathic ability.^8^^,^^15^^,^^16^^,^^34^ It was argued by our participants that surgeons were less empathic than their peers in other specialties, and that the conditions of their working practice (quick ward rounds before theatre lists, shorter lengths of stay and therefore less opportunity to build doctor-patient relationships) created a working culture where there was little empathy towards patients. Conversely, clinicians working in specialties where patient contact was greater, such as paediatrics and psychiatry, were perceived to have higher empathic ability. Some Foundation doctors felt that they were under pressure to conform to the cultural practice of a specialty, and that this had compromised both their opportunity to empathise, and the patient’s care.

The theme of doctors’ value judgements of patients and their illnesses also emerged strongly from the data. Some participants found it difficult to empathise with patients diagnosed with illnesses perceived to be self-inflicted, in contrast to those thought to have acquired their disease through ill fortune. Further value judgements were made against patients who appeared to have psychosomatic symptoms, opiate addiction and alcoholism, and thus were denied the opportunity to receive empathy from their doctor. The idea that it is easier to empathise with a patient similar to oneself also emerged. In the context of a rapidly ageing and ethnically diverse population, this is particularly problematic.

### Perceived value of empathy in clinical practice

Despite these concerns, many participants valued empathic ability as a desirable characteristic. Those clinicians able to empathise were considered to be excellent communicators. In addition, empathising with patients was seen as an important component of holistic care, and in some cases may be the intervention most likely to increase patient satisfaction. However, some participants considered that an excess of empathy or deeper emotional engagement with patients might impede objectivity, leading to poor clinical judgement, or increasing levels of clinician stress and anxiety. Balancing empathic ability with objective clinical judgement seemed to be valued by many Foundation doctors.

### Implications for medical educators

Currently, there is little evidence to evaluate the hypothesis that empathy skills can be taught to doctors in training. Studies reporting an increase in empathy amongst medical undergraduates exposed to the humanities (particularly literature) are interesting but limited,^35^^,^^36^ and the practical application of that idea may be challenging, given the demands of modern curricula. Similarly, the development of narrative competence, where individuals engage with literature and practise reflective writing, has been suggested as a model for humane medicine.^37^ This study found that most Foundation doctors perceive that empathy skills are acquired during childhood, usually as a consequence of parental influence. Some doctors believed that empathy skills could be refined by training, but many found the methods employed during such training to be trite, disingenuous and ultimately of low value. Accordingly, there may be a role for novel teaching methods such as Schwartz Rounds,^38^^,^^39^ where patients have the opportunity to describe their lived experiences to clinical staff. There is tentative data to support their use in reducing clinician stress, increasing compassion, and developing confidence in attending to the psychosocial needs of patients.^40^

## Conclusions

The perceived decline in empathy amongst UK Foundation trainees is concerning, particularly in the context of the Francis Report. Importantly, most participants reported a conscious acknowledgement of this decline, and considered it to be secondary to the myriad physical and emotional demands of working as a newly-qualified doctor. Some participants reported that they felt they could only afford to give secondary consideration to patients’ emotional and psychological needs, as they prioritised physical well-being. This was perceived most commonly in surgical specialties, where the rapid turnover of patients and the working culture may inhibit empathy and the development of meaningful doctor-patient relationships. In addition, some participants perceived that their ability to deliver empathy was influenced by their value judgements of the patient and their illness, as well as the patient’s socio-cultural similarity to the doctor’s friends and family.

The challenge for medical educators is how to address these issues in a modern healthcare system. Shorter lengths of hospital stay, greater numbers of patients, and an increasingly aged, obese and culturally diverse population may accelerate empathy decline, ultimately risking poor clinical care. This study reports the perceptions of ethical erosion amongst UK Foundation doctors, but there is a need for further study of educational strategies that may be useful in reversing this trend.

### Conflict of Interest

The authors declare that they have no conflict of interest.
